# The pLysRS-Ap_4_A Pathway in Mast Cells Regulates the Switch from Host Defense to a Pathological State

**DOI:** 10.3390/ijms22115620

**Published:** 2021-05-25

**Authors:** Sharmila Govindaraj, Lakshmi Bhargavi Paruchuru, Ehud Razin

**Affiliations:** Department of Biochemistry and Molecular Biology, Institute for Medical Research Israel-Canada, Faculty of Medicine, The Hebrew University of Jerusalem, Jerusalem 91120, Israel; sharmila.govindaraj@mail.huji.ac.il (S.G.); bhargavi.lakshmi@mail.huji.ac.il (L.B.P.)

**Keywords:** allergic disease, mast cell activation, host defense, pathological condition, pLysRS-Ap_4_A signaling

## Abstract

The innate and adaptive immune systems play an essential role in host defense against pathogens. Various signal transduction pathways monitor and balance the immune system since an imbalance may promote pathological states such as allergy, inflammation, and cancer. Mast cells have a central role in the regulation of the innate/adaptive immune system and are involved in the pathogenesis of many inflammatory and allergic diseases by releasing inflammatory mediators such as histamines, proteases, chemotactic factors, and cytokines. Although various signaling pathways are associated with mast cell activation, our discovery and characterization of the pLysRS-Ap_4_A signaling pathway in these cells provided an additional important step towards a full understanding of the intracellular mechanisms involved in mast cell activation. In the present review, we will discuss in depth this signaling pathway’s contribution to host defense and the pathological state.

## 1. Mast Cells (MCs) and Their Activation Mechanisms

Mast cells (MCs) are a subset of leukocytes and are derived from hematopoietic progenitor cells. In 1879, Paul Ehrlich characterized the mast cell by the presence of large granules in the cytoplasm and identified them as “*mastzellen*”, meaning “feeding cells”. Mast cells (MCs) are sentinel cells of connective tissue that, upon activation by an allergen, can trigger an immediate inflammatory response. An immature and undifferentiated form of mast cells circulates in blood and migrates to tissues of the body that are in close proximity with the external environment, such as skin, airways and intestine. Maturation of mast cells (MCs) occurs in these vascular tissues aided by cytokines, stem cell factors secreted by fibroblasts and endothelial cells [1].

There are several mechanisms of mast cell activation, but the most important signaling pathway is the antigen–allergen interaction with the immunoglobulin E (IgE) antibody in the tissues and the subsequent IgE binding to the high-affinity receptor Fc epsilon RI (FcεRI). This IgE-FcεRI cross-linking triggers multiple transduction pathways allowing the mast cells (MCs) to secrete their various allergic mediators (histamines, heparins, proteoglycans, neutral proteases, and cytokines) to the external environment. Another type of immunoglobulin and Fc receptor triggering also assists the host defense in response to Interferon (IFN) during the condition of an autoimmune disease, an *IgG-FcγRI* cross-linking causes the human mast cell (MC) activation [2]. Their Toll-like receptors (TLRs) are surface pathogen recognition receptors (PRRs) that can directly activate mast cells and help in controlling bacterial infections [3]. In addition to microbial challenges, an anti-viral response mechanism in rat peritoneal mast cells (MCs) has been described in which viral dsRNA binding to TLR3 can trigger the release of IFN-*α* and *β*, various chemokines and cytokines such as CCL3, CXCL8, TNF, and IL-1*β*, and a pro-inflammatory lipid mediator, cysLTs [4].

Mast cells (MCs) are one of the major effector cells in vascular connective tissues with a key role in the regulation of innate and adaptive immunity. They are involved in the pathogenesis of many inflammatory and allergic diseases, such as immediate hypersensitivity autoimmune diseases [5], experimental allergic encephalomyelopathy [6], rheumatoid arthritis [7], as well as in delayed-type hypersensitivity [8], tumor growth, angiogenesis [9] and congestive heart failure [10]. Under physiological conditions, the host defense mechanism of mast cells against the invading pathogens is due to the increased IgE-FcεRI cross-linking. The hypersensitivity of mast cells will switch the host defense to a pathological condition ranging from simple nasal allergies caused by pollen or fungal spores to mild dermatological eczema/erythema and to severe asthma or hypoxia, leading to life threatening anaphylactic allergic reactions [11].

## 2. The pLysRS Signaling Pathway in IgE-FcεRI Activated Mast Cells (MCs)

The FcεRI receptor is found on various immune cells such as mast cells (MCs) and basophils, and is a multimeric protein that comprises an external IgE-binding α subunit, a transmembrane β subunit and a dimer of disulfide-linked γ subunits [12]. Cross-linking of FcεRI with an IgE bound antigen results in the triggering of a series of complex signal transduction pathways as a part of the allergic response of mast cells (MCs). The immunoreceptor tyrosine-based activation motifs (ITAMs), located on the γ subunit of FcεRI, provide a docking site for Syk after being tyrosine-phosphorylated by Lyn and Src kinases. The enzymatically activated Syk sends a downstream signal and phosphorylates several proteins, including phospholipases Cγ (PLCγ), phosphoinositide 3-kinases (PI3Ks), regulatory subunits and several other SH2 domain-containing leukocyte protein (SLP) family proteins [13]. Various small GTPases, such as Rac, Ras and Rho, are also activated; thus, causing the stimulation of the ERK, JNK and p38 MAP kinase pathways [14]. Here, MAP kinases play a crucial role in regulating the translocation of a variety of transcription factors to the nucleus from the cytosol, thereby controlling the degranulation process. It was identified from our previous works that IgE-antigen stimulation induces serine 207 phosphorylation of Lysine-tRNA synthetase (LysRS) by this MAPK-dependent stimulation, allowing the pLysRS to be released from the multi-synthetase complex (MSC) [15].

## 3. pLysRS Transcriptional Regulation of MITF, USF2 via the Ap_4_A–HINT Pathway

**Figure 1:**
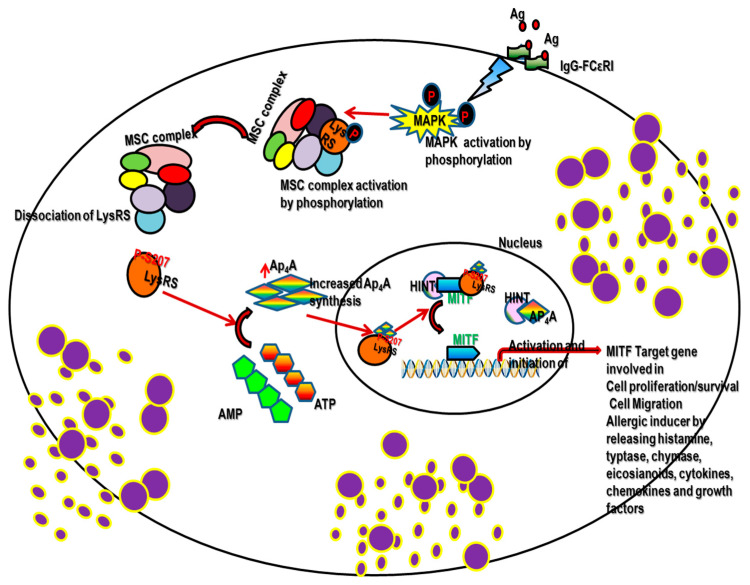
Demonstrates the schematic diagram of pLysRS-Ap_4_A signaling in mast cell activation. Our pioneering work demonstrated, following physiological stimulation in the mast cell, the activated ERK-mediated phosphorylation of 207 serine lysyl-tRNA synthetase (LysRS), which is one of the components of the multi-synthetase–cytosolic complex. This confirmational change in the LysRS enzyme causes its dissociation from its complex and catalysis in the synthesis of Ap4A. This Ap4A acts as a 2nd messenger and binds specifically to Hint1 in the nucleus. This change in confirmation causes its dissociation from the transcription factor MITF, which is now able to activate its target genes of MITF that are involved in cell proliferation, migration, allergic inducer, etc.

LysRS is a member of the aminoacyl-tRNA synthetase (aaRSs) family, and has been shown to be involved not only in the translation step of protein synthesis, but also as a transcriptional regulation factor of MITF and USF2 [16,17]. In mammalian cells, LysRS forms a cytosolic aminoacyl-tRNA MSC along with another eight aaRSs and three nonanalytic components, p18, p43 and p38 [18]. When mast cells (MCs) are in the quiescent state, LysRS is bound to the cytoplasmic MSC via its interactions with p38. In the activated mast cell (MC), a series of MAPK signaling cascades lead to phosphorylation of LysRS at Ser207 to give LysRSPS207, which is essential for Ap_4_A production, and the pLysRS being released from the MSC. Mutation of serine at LysRSS207 to alanine (LysRSS207A), which mimics the dephosphorylation state, significantly reduced Ap_4_A production. In contrast, over expression of the phosphomimetic mutant LysRSS207D (serine mutated to aspartate) increased the concentration of Ap_4_A; thus, enhancing the transcription of MITF target genes in mast cells (MCs) [15]. In both prokaryotes and eukaryotes, Ap_4_A provides an important signal for gene expression, and pLysRS’s involvement in Ap_4_A production through a zinc-dependent chemical reaction has been known for decades [19,20]. The identification of the pLysRS-Ap_4_A–MITF signaling pathway by our group demonstrated that Ap_4_A acts as a secondary messenger whereby, as a result of the IgE-antigen stimulation, Ap_4_A binds to the histidine triad nucleotide-binding protein 1 (HINT1), causing the release of MITF from the complex. We identified by Biacore analysis that specific binding of HINT1 to Ap_4_A, but not to other Ap_n_A molecules (Ap_3_A or Ap_5_A), affected the MITF–HINT-1 protein complex [17]. A similar mechanism was identified in USF2, another transcription factor, whereby USF2 is regulated by the interaction of HINT1 with Ap_4_A [16]. Thus, pLysRS regulates the MITF and USF2 transcriptional activity via Ap_4_A as illustrated in Figure 1.

## 4. Role of pLysRS-Ap_4_A Mediated Signaling under Pathological Conditions

The location of mast cells at the host–environment interface generates inflammatory reactions by releasing pro-inflammatory mediators during the immune activation [21] and a disordered homeostasis causes multiple pathological conditions and could result in mast cell activation syndrome [22]. It has been identified from many studies that mast cells provide the first line of defense against the invading pathogens through multiple mechanisms. Activated mast cells (MCs) functionally impact the other immune cells’ activities by their effective signaling mechanism; then, control the pathological conditions caused by autoinflammatory cells, cancerous cells or by external invaders such as bacteria and viruses.

## 5. Autoimmune Disease

Previous studies on multiple sclerosis and rheumatoid arthritis recognized the significant role played by mast cells (MCs) in the initiation and propagation of these autoimmune diseases. The presence of various mast cell (MC) mediators, such as tryptase, in the cerebrospinal fluid of multiple sclerosis patients [23] supports the inflammatory response by activating peripheral mononuclear cells to produce various factors, including TNF, IL-6, and IL-1; then, stimulates the protease-activated receptor (PAR) causing microvascular leakage [24]. Although various cytokines and mediators of mast cells (MCs) were identified in the regions of inflammation in autoimmune conditions, the necessity of LysRS-Ap_4_A signaling molecules remains unclear. Cyclic GMP–AMP (cGAMP) synthetase (cGAS) belongs to the family of intracellular PRRs. It identifies cytosolic soluble stranded nucleic acids (dsDNAs) and generates cGAMP, which in turn binds to the stimulator of the interferon genes (STING). This cGAS–STING pathway produces type 1 IFNs and NF-κB necessary for the induction of the pro-inflammatory response, and a tight regulation is needed for the suppression of autoimmune diseases [25]. Recently, LysRSAp_4_A were proposed as pharmacological targets to control the STING signaling pathway for the treatment of autoinflammatory diseases. Of the two mechanisms identified, one showed that LysRS interacts with RNA–DNA hybrids, causing the delay in recognition by cGAS essential for cGAMP production. In the other mechanism, LysRS-dependent Ap_4_A production causes the attenuation of STING signaling; thus, regulating the autoimmunity disease [26].

## 6. Tumor Microenvironment

The tumor microenvironment plays a pivotal role in cancer progression and metastasis. As a result of chronic inflammation, the tumor microenvironment frequently contains an abundance of growth factors, cytokines, chemokines, prostaglandins, and reactive oxygen species and also immune cells such as mast cells (MCs), tumor-associated macrophages (TAMs), myeloid-derived suppressor cells (MDSCs), as well as adaptive immune cells, including T and B lymphocytes and dendritic cells (DCs) which can promote tumor metastasis.

In recent years, the involvement of cytosolic aaRSs in tumorigenesis and metastasis, apart from their canonical (protein translation) role [27], has been the subject of much research. Of the aaRSs, LysRS is highly expressed in various tissues such as the colon, lung, gastric, breast and thyroid cancer cells, and has noncanonical functions in the immune response and tumor metastasis [28]. The activation of the pLysRS-Ap_4_A-regulated MITF pathway is the main hallmark in both cancer and immune cells. An early report from our group described how activated serine 207 phosphorylated lysyl-tRNA synthetase (p-s207 LysRS) is released from the cytoplasmic MSC; then, relocates into the nucleus, promoting the transcriptional activity of MITF in stimulated cultured mast cells and cardiomyocytes. The activation of MITF is mediated by an increased Ap_4_A level through the release of HINT1 suppression [16,17,29,30,31,32]. Since LysRS is found in all tissues, the activation of the p-LysRS-Ap_4_A pathway occurs in cancer cells also. The HINT1 (tumor suppressor protein)-deficient mice have an increased susceptibility for both spontaneous tumors and tumor proliferation [33]. Hence, this pathway should be tightly regulated. Ap_4_A regulates MITF activation through HINT1 polymerization. The polymerization of HINT1 may block the MITF–HINT1 interaction that releases MITF for the increased transcription in the MITF downstream genes (*c-Met*, *c-Kit*). Thus, the second messenger Ap_4_A activates the MITF downstream target by mediating HINT1 polymerization that is involved in cell proliferation and migration [34]. Phosphorylation of S207 on LysRS by ERKs results in translocation to the nucleus, and is associated with an improved mean disease-free survival (DFS) in patients with EGFR mutations [35] compared to WT EGFR patients. This was also followed by the participation of membranous LysRS and nuclear LysRS in invasive cell dissemination—an important step in cancerous cell progression was identified in colon cancer spheroids [36].

**Figure 2:**
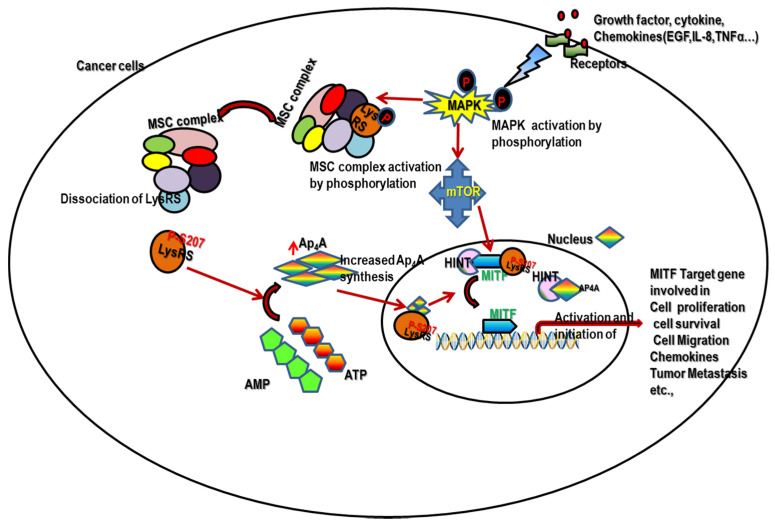
Represents the schematic diagram of pLysRS-Ap_4_A signaling activation during cancer metastasis. During chronic inflammation, the immune cells, such as mast cells (MCs), tumor-associated macrophages (TAMs), etc., release an abundance of growth factors, cytokines, chemokines, prostaglandins, and reactive oxygen species in the tumor microenvironment which can promote tumor metastasis. The released growth factors, chemokines and cytokines cause the activation of the pLysRS-Ap_4_A-regulated MITF pathway. Thus, it promotes cancer cell survival, proliferation, migration and metastasis. Hence, the pLysRS-Ap_4_A-regulated MITF pathway is the main hallmark in both cancer and immune cells.

DCs are involved in acquired immune activity by migrating to lymph nodes for antigen presentation to naive T or B cells [37]. The highly conserved function of antigen presentation can be precisely impaired by low levels of intracellular Ap_4_A. Our previous study shows that DCs may functionally benefit from the increased intracellular Ap_4_A concentration by knocking out Ap_4_A hydrolase, thereby enhancing the antigen cross-presentation by DCs [38], which aids in antigen recognition by immune cells during cancer conditions. The above evidence clearly indicates that p-LysRS-Ap_4_A-regulated MITF signaling pays a major role in tumor metastasis (Figure 2).

## 7. Conclusions

The delineation of a new signaling mechanism could provide new insights and a better understanding of a disease condition. The discovery and characterization of the p-LysRS-Ap_4_A signaling pathway has attracted the attention of many researchers. pLysRS has several roles besides its function as a key enzyme involved in translation. In the immune system, it may function both as an extracellular cytokine-like molecule and a signal transduction protein in a signal transduction pathway, ultimately regulating gene expression. Apart from its canonical mechanism, the p-LysRS-Ap_4_A-regulated MITF pathway is involved not only in allergic disease but also in cancer metastasis. It seems that much more research is needed in order to understand the complex regulation of the pLysRS function and its involvement in such various unrelated pathological processes as described above. Such understanding could help in designing potential drug targets for various pathologies.

## Data Availability

Not applicable.

## Abbreviations

aaRSs	Aminoacyl-tRNA synthetase
Ap3A or Ap5A	Diadenosine tetraphosphate or diadenosine pentaphosphate
Ap4A	Diadenosine tetraphosphate
B cell	B cell lymphocytes
CCL3	Chemokine CC motif ligand 3,
cGAMP	Cyclic GMP–AMP
cGAS	Cyclic GMP–AMP(cGAMP) synthetase
*c-Kit*	CD117, also called KIT or c-Kit receptor
*c-Met*	Mesenchymal–epithelial transition factor
CXCL8	Chemokine CXC motif ligand 8
DCs	Dendritic cells
EGFR	Epidermal growth factor receptor
ERK	Extracellular signal-related kinase
HINT1	Histidine triad nucleotide-binding protein 1
IFN-*α*, *β*	Interferon alpha, beta
IFNγ	Interferon gamma γ
IgE	Immunoglobulin E
IgE-FcεR	Immunoglobulin E FC epsilon RI
IgG-FcγRI	Immunoglobulin G FC gamma RI
IL-1	Interleukin-1
IL-1*β*	Interleukin-1 beta
IL-6	Interleukin-6
ITAM	Immunoreceptor tyrosine-based activation motifs
JNK	Janus kinase
Lyn	Lck/Yes novel tyrosine kinase
LysRS	Lysine-tRNA synthetase
LysRSS207A	Lys phosphorylation at serine 207 serine-to-alanine mutation
LysRSS207D	Lys phosphorylation at serine 207 serine-to-aspartate mutation
MAPK	Mitogen-activated protein kinase
MCs	Mast cells
MDSCs	Myeloid-derived suppressor cell
MITF	Microphthalmia-associated transcription factor
MSC	Multisynthetase complex
NF-κB	Nuclear factor kappa B
p38 MAP kinase	p38 mitogen-activated protein kinase
PAR	Protease-activated receptor
PI3Ks	Phosphoinositide 3-kinases
PLCγ	Phospholipases Cγ
pLysRS	Phosphorylated lysine-tRNA synthetase
PRRs	Pattern recognition receptors
Rac	Rho family GTPase
Ras	Rat sarcoma
Rho	Ras homologous protein
SLP	SH2-domain-containing leukocyte protein
Src	v-src sarcoma (Schmidt-Ruppin A-2) viral oncogene homolog (avian)
STING	Stimulator of interferon genes
SyK	Spleen tyrosine kinase
T cell	Thymus lymphocytes
TAMs	Tumor-associated macrophages
TNF	Tumor necrosis factor
USF2	Upstream transcription factor 2
